# Acute Paediatric Tracheal Deviation and Neck Lump Secondary to Food Bolus Impaction

**DOI:** 10.7759/cureus.21553

**Published:** 2022-01-24

**Authors:** Quentin Bonduelle, John Yaro, Youssef Aladham, Mark Johnston

**Affiliations:** 1 Otolaryngology, University Hospitals of Leicester, Leicester, GBR; 2 Otolaryngology, University Hospitals of Derby and Burton, Derby, GBR

**Keywords:** neurodevelopmental delay, food bolus, tracheal deviation, neck lump, paediatric age

## Abstract

Acute presentations of paediatric tracheal deviation secondary to neck masses are rare. The differentials are broad and the child may be compromised. Stabilising and resuscitating the child are the primary aims.

This case describes a six-year-old boy with a history of neurodevelopmental delay and progressive dysphagia, presenting with an acute history of soft food bolus impaction, significant tracheal deviation and a firm neck lump. We discuss the diagnostic difficulties of the presentation, the work-up and the management of this rare case in the setting of a university hospital in the United Kingdom, with no paediatric intensive care on site.

## Introduction

Lateral deviation of the trachea may be a normal finding on a paediatric chest X-ray, but it could well represent an airway emergency. This requires a rapid diagnosis to establish a surgical or medical treatment plan. Differential diagnosis of pathological tracheal deviation includes tension pneumothorax, upper lobe lung collapse and cervical or mediastinal masses. Acute presentations of paediatric tracheal deviation secondary to neck masses are rare. The differentials are broad and the child may be compromised. Stabilising and resuscitating the child are the primary aims [1].

This case describes a six-year-old boy with a history of neurodevelopmental delay and progressive dysphagia, presenting with an acute soft food bolus impaction, significant tracheal deviation and a firm neck lump.

## Case presentation

A six-year-old boy was brought to our emergency department (ED) by his mother. He had absolute dysphagia since eating meatballs earlier that day. His past medical history included birth at 25 weeks gestation with subsequent neurodevelopmental delay and associated dysphagia; the patient was normally on a pureed diet.

**Figure 1:**
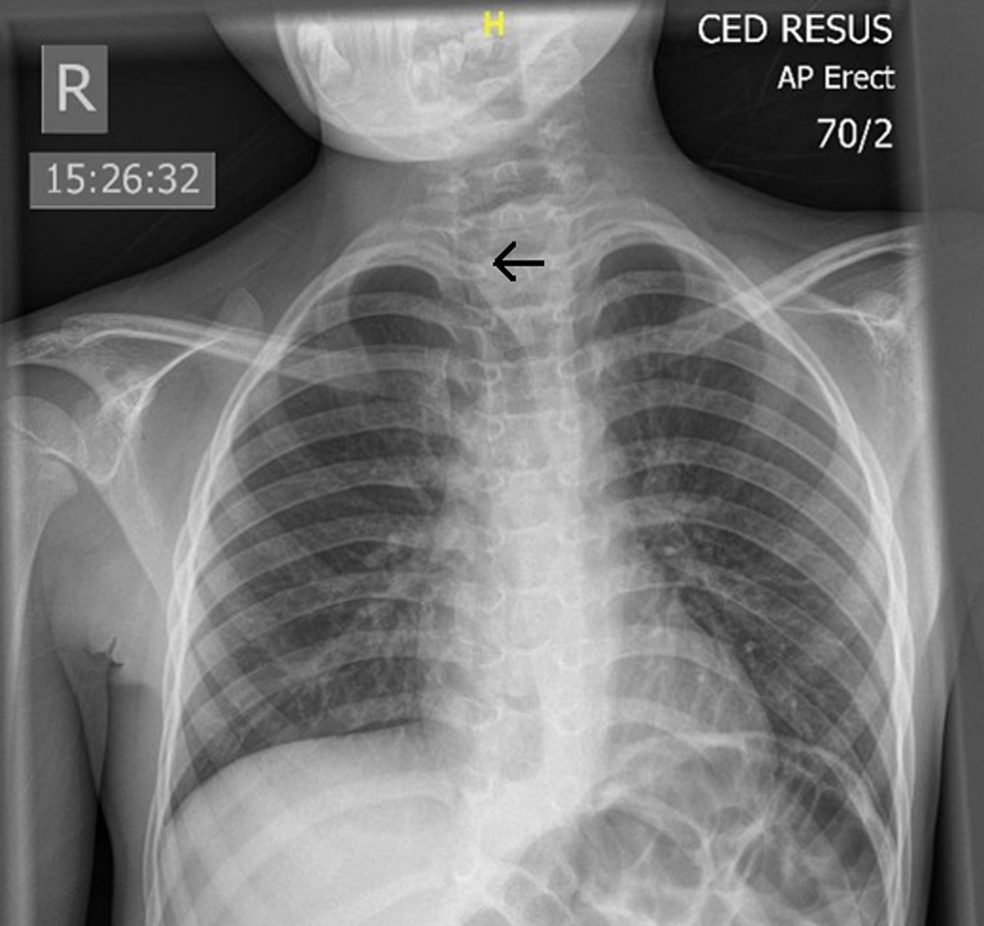
Chest X-ray Admission anterior-posterior erect chest X-ray demonstrating a deviated trachea to the level of the right sternoclavicular joint (arrow) and widened superior mediastinum.

On arrival, his pulse rate was 82 beats/minute, blood pressure was 125/90 mmHg and respiratory rate was 16/minute. A chest X-ray (CXR) was performed (Figure 1). The child was desaturated whilst in the department, requiring transfer to the paediatric resuscitation room on 15 L/min of oxygen via a non-rebreathe mask to maintain oxygen saturations >94%.

Whilst the anaesthetic team were called and the patient was stabilised, a focussed history and examination were performed. A history of progressive dysphagia became apparent, worse with bread and meat. The child was on a pureed diet at school but had eaten quartered meatballs earlier in the day. The child was otherwise well, and there were no head and neck red flags or systemic symptoms. The mother was unaware of any neck lumps previously.

On examination, there was a significant right-sided tracheal deviation to the right sternoclavicular joint and a firm 4 x 4 cm non-pulsatile mass in level 4 of the left side of the neck (between the inferior margin of the cricoid and the clavicle). There were no associated superficial skin changes. The child had intermittent respiratory distress and lots of secretions, but no stridor. Admission blood tests were normal and the CXR demonstrated superior mediastinal widening with significant tracheal deviation to the right. There were no radio-opaque foreign bodies, lung changes or signs of perforation (Figure 1).

After discussion and consent with the child’s mother, he was taken to theatre for intubation, ventilation, removal of a suspected food bolus and subsequent transfer for computed tomography (CT) to further investigate the superior mediastinal mass. He was gas-induced with sevoflurane and kept spontaneously breathing throughout. The child was intubated using a Macintosh laryngoscope and a size 5 endotracheal tube (ETT), mounted over a flexible bronchoscope. The laryngeal inlet was displaced and rotated to the right. No foreign body was found in the airway down to the carina. There were copious secretions with food particles in his upper aerodigestive tract. A rigid oesophagoscopy was performed, identifying impacted meatballs at 15 cm from the incisors. These were mobilised distally to 25 cm from the incisors, after significant debulking with graspers and attempted retrieval using graspers, forceps and a Dormia basket. In view of the distance of the food bolus and our equipment available, the remaining meatball was left in situ. The oesophageal mucosa was intact on inspection and the dentition unchanged but noted to be poor, with multiple missing teeth. No abnormality was noted in the oesophagus. Interestingly, the trachea was central and the neck lump absent on examination after the oesophagoscopy, which raised the suspicion that the clinical findings were related to the soft food bolus.

**Figure 2:**
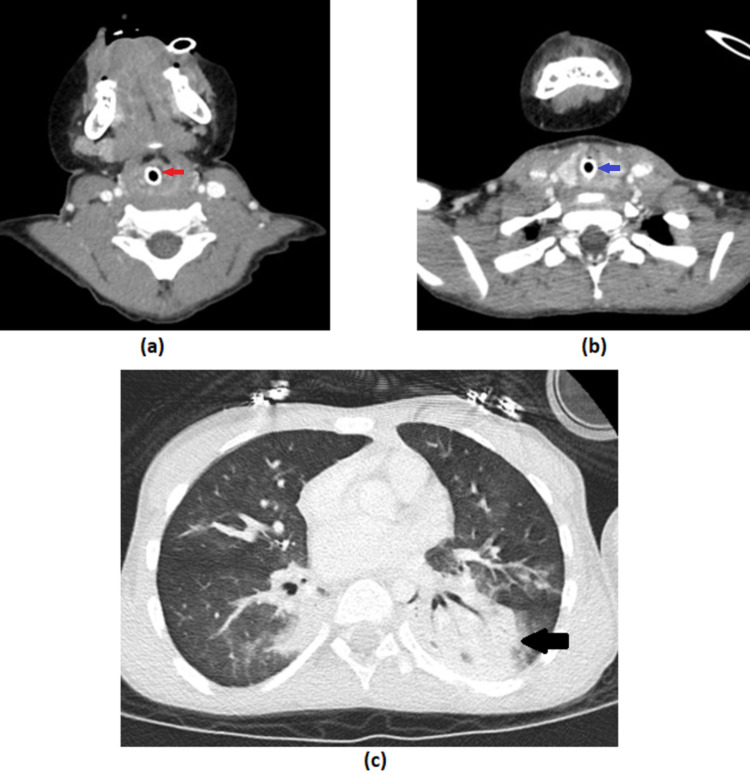
Computed tomography Axial plane CT scan with contrast whilst the patient was intubated. Images (a) and (b) demonstrate midline larynx (red arrow) and trachea (blue arrow) and absence of lesions, masses or vascular abnormalities in the neck. Image (c) demonstrates aspiration with collapse/consolidation of the lower lobes (left > right) (black arrow).

A CT scan of the neck and chest with contrast was performed following tracheo-oesophagoscopy, confirming these suspicions. The neck was normal with no masses or signs of perforation (Figure 2). However, both lower lobes showed signs of collapse and consolidation, in keeping with aspiration.

**Figure 3:**
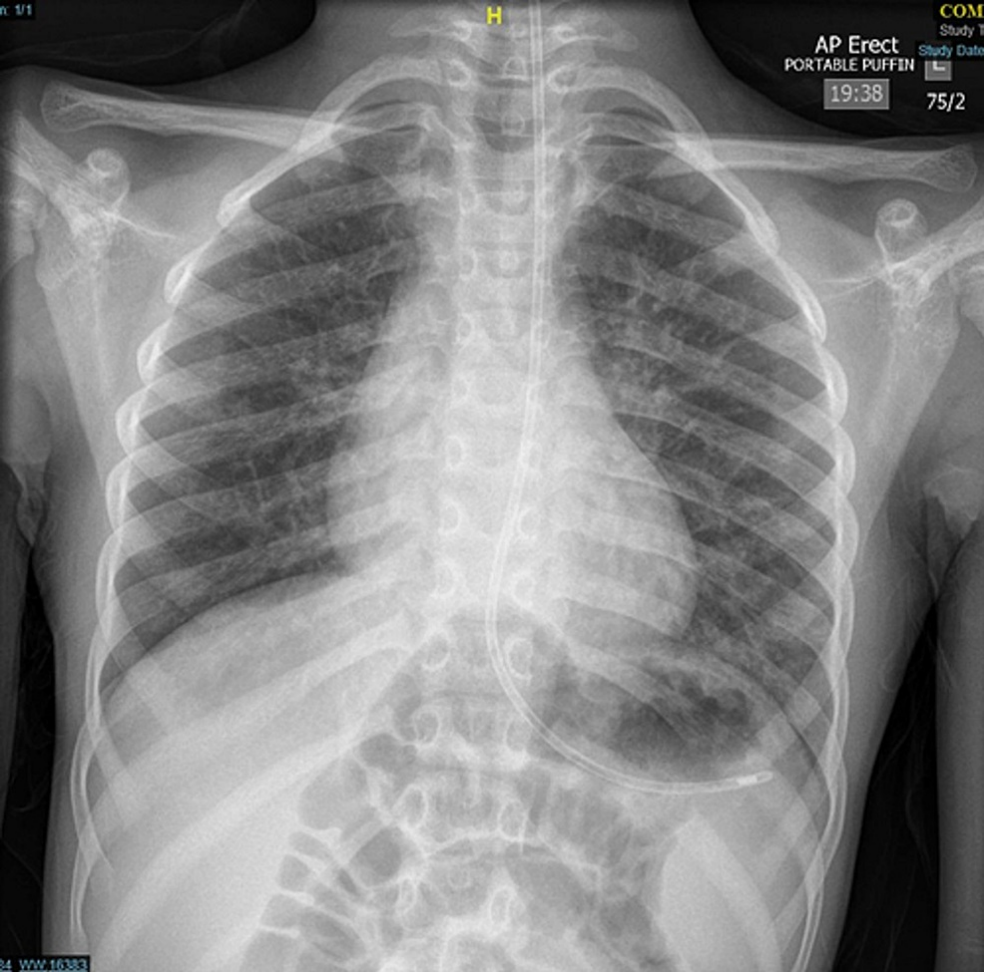
Postoperative chest X-ray Postoperative chest X-ray showing midline trachea and resolution of the widened mediastinum, with a nasogastric tube in situ.

The child was transferred to the local tertiary hospital paediatric intensive care for ongoing ventilatory support and further investigations. The child was extubated the subsequent day, treated for aspiration pneumonia with a course of antibiotics, had a period of nasogastric (NG) feeding and no abnormalities were found on flexible oesophagogastroscopy performed four weeks later. A repeat CXR prior to discharge showed a midline trachea and resolution of the widened superior mediastinum (Figure 3). A feeding plan was decided by the speech and language team and dietitians, with parental involvement, prior to discharge.

## Discussion

Neck lumps causing significant tracheal deviation are very rare in children. The tracheal deviation is caused by extraluminal pressure effects. The underlying pathology originates from structures within the neck, mediastinum or hemithoraces. It may also be a normal variant in children up to five years of age when the trachea is more flexible [2]. This case demonstrates tracheal deviation caused by a soft food bolus impacted within the cervical oesophagus, presenting as a neck lump and a widened superior mediastinum on CXR. The child had a history of dysphagia, which is seen in up to 80% of children with neurodevelopmental delay [3]. Our primary concern with this desaturating child was securing the airway and optimising ventilation. He was too unwell to be retrieved by the Children Medical Emergency Transport (COMET) team and transferred to the nearest tertiary centre. In view of the absence of stridor and a discussion with the on-call thoracic surgeon, the deviated trachea was deemed to be intubatable. Although CT is the imaging of choice to evaluate the trachea, the CXR was sufficient to determine tracheal patency (Figure 1) [4].

Initial management decisions and safe transfer to theatre relied on clear communication to the parent and amongst the multidisciplinary team: anaesthetist, otolaryngologist, paediatric emergency team and theatre team. By mounting an age-appropriate ETT over a flexible bronchoscope, the airway was examined whilst intubating the child. Alternative methods include using a ventilating bronchoscope or a laryngoscope (e.g. Lindholm), with an ETT mounted on a rigid 0^o^ endoscope. Once the airway was secured and the child was ventilating well, we proceeded to manage the soft food bolus, being careful in view of a history and examination findings suggestive of a potential structural underlying cause. A variety of techniques can be used for soft food boluses, broadly classified into pushing or extraction techniques [5].

Our differential diagnoses included oesophageal masses, thyroid masses, thymus masses, lymphoma and vascular abnormalities. The CT with contrast was performed to exclude these. Reassuringly, the cause of the lump and tracheal deviation was the meatball food bolus, as demonstrated with post-oesophagoscopy examination and imaging (Figures 2, 3). This case demonstrates that food bolus impaction should be considered as a differential diagnosis for acute presentation of neck lump and tracheal deviation, particularly in the context of neurodevelopmental delay associated dysphagia and patulous oesophagus. The paediatric, speech and language therapist (SLT) and dietitian teams are instrumental in optimising safe long-term feeding in such cases, with active parental involvement.

## Conclusions

Soft food bolus impaction can present with a neck lump and tracheal deviation in children. The child may be compromised and may have risk factors for developing soft food bolus impaction. Simultaneous assessment and resuscitation is the primary focus in an unwell child. Prompt exclusion of life-threatening differential diagnosis is paramount in a deteriorating child with tracheal deviation and a neck lump. Patient safety is the primary concern and transfer to an appropriate centre, if the patient is stable enough, should be considered. Once the airway is secured and the patient is stabilised, pushing and extraction techniques are implemented through oesophagoscopy to dislodge impacted food boluses. Further investigations including cross-sectional imaging of the neck and thorax and endoscopy may be required to exclude underlying causes. Dysphagia is a common symptom in children with neurodevelopmental delay and is best managed by the multidisciplinary team, with input from the paediatric doctors, speech and language therapy team and dietitian team.
